# Brief localised monocular deprivation in adults alters binocular rivalry predominance retinotopically and reduces spatial inhibition

**DOI:** 10.1038/s41598-020-75252-w

**Published:** 2020-10-30

**Authors:** Shui’er Han, David Alais, Hamish MacDougall, Frans A. J. Verstraten

**Affiliations:** 1 School of Psychology, University of Sydney, Sydney, Australia; 2 Institute for Infocomm Research, Agency for Science, Technology and Research, Singapore, Singapore

**Keywords:** Neuroscience, Psychology

## Abstract

Short-term deprivation (2.5 h) of an eye has been shown to boost its relative ocular dominance in young adults. Here, we show that a much shorter deprivation period (3–6 min) produces a similar paradoxical boost that is retinotopic and reduces spatial inhibition on neighbouring, non-deprived areas. Partial deprivation was conducted in the left hemifield, central vision or in an annular region, later assessed with a binocular rivalry tracking procedure. Post-deprivation, dominance of the deprived eye increased when rivalling images were within the deprived retinotopic region, but not within neighbouring, non-deprived areas where dominance was dependent on the correspondence between the orientation content of the stimuli presented in the deprived and that of the stimuli presented in non-deprived areas. Together, these results accord with other deprivation studies showing V1 activity changes and reduced GABAergic inhibition.

## Introduction

Ocular dominance is the tendency for visual cells in early cortex to prefer input from one eye^1^. This preference is plastic and can be very sensitive to disturbances in early visual development^2–4^. This was first demonstrated in 1963 by Wiesel and Hubel, who sutured an eye of a kitten for a period of three months to examine the effects of monocular deprivation on primary visual neurons. Upon re-opening the deprived eye, the authors found a shift in neural response favouring the non-deprived eye, a shift which persisted indefinitely despite the newly opened eye receiving equivalent visual input to the other eye. A follow-up study repeated the deprivation procedure on an adult cat for an even longer period of 1 year^5^ but found no shift in ocular dominance in the mature cat. These findings contributed to the idea of a *sensitive period* in development during which visual cortical plasticity occurs and beyond which changes are limited^6^ and require longer periods of deprivation to effect long-term changes^7^.

More recent research revealed a greater degree of plasticity in human ocular dominance than previously thought. Using a binocular rivalry (BR) paradigm^8,9^ in young adults, patching an eye with a translucent patch that excludes pattern information but not mean luminance for 2.5 h produced a temporary boost of the eye’s dominance in a binocular rivalry task^10^. This counterintuitive boost is termed the deprivation aftereffect (DE) by Blake and colleagues^11^, and it has been measured in a number of assessment tasks apart from BR. Examples include dichoptic masking^12^, dichoptic global motion coherence^13^ and binocular combination^13,14^, though it is worth noting that the type of pattern deprivation (e.g., presenting phase scrambled images to the deprived eye) and assessment task may affect the magnitude of increase observed^15,16^. Other studies have explored alternative monocular deprivation methods, finding that complete monocular occlusion^13^, a monocular kaleidoscopic lens^17^, dichoptic movies^15^, continuous flash suppression (CFS)^11,18^ and interocular contrast differences^18^ were capable of producing similar boosts to the deprived eye. Thus, the DE does not depend on monocular pattern deprivation, but on the differential stimulation of the eyes.

While most studies interested in the effects of short-term monocular deprivation have typically employed hours of monocular deprivation, e.g., 2.5 h^10,12–17^, two recent studies have shown that the DE can be obtained with shorter deprivation durations. For example, Min et al. discovered that the interocular contrast balance ratio on a binocular combination task varies little with deprivation durations, producing only a 25% increase in the DE for a 300-min deprivation period compared to a 15-min deprivation period^19^. Similarly, Kim et al. found that 15 min of CFS exposure was capable of producing significant shifts in BR dominance that temporarily strengthened the deprived eye^18^. These shifts were observed in predominance (i.e., percentage of total viewing time where an eye is dominant) and mean dominance duration, but the changes in the former measure were longer-lasting, presumably because it is a more sensitive measure for the weaker effects obtained with brief deprivation periods. In line with this idea, the authors also reported significant shifts in predominance after only 3 min of continuous flash suppression or interocular contrast difference.

Prior exposure to a high contrast visual pattern typically reduces the detection sensitivity to that pattern and similar pattern structures^20,21^. It is therefore plausible that the extended presentation of higher contrast stimuli to one eye could have reduced its sensitivity relative to the other (deprived) eye, thereby decreasing the adapted eye’s dominance in the immediate post-deprivation period. A more common (but not exclusive) interpretation is homeostatic plasticity, where the period of reduced stimulation triggers an upregulation of contrast gain in the deprived eye^10^. This upregulation could be executed through contralateral inhibition, which modulates the gain of each eye prior to binocular combination^22^. Consistent with this idea, Spiegel and colleagues^23^ reported a lower incidence of binocular fusion and increased suppression of the non-deprived eye, both of which were counteracted by an interocular contrast difference. Studies also reported an increase in V1 activity corresponding to the deprived eye^24,25^, which interestingly, occurred mainly in the parvocellular pathway^25^. A similar bias was reported with psychophysical observations, where longer lasting DEs were observed with chromatic BR gratings^26^. While these observations do not necessarily rule out contrast adaptation, they accord with the greater plasticity potential of the parvocellular pathway^27,28^.

**Figure 1:**
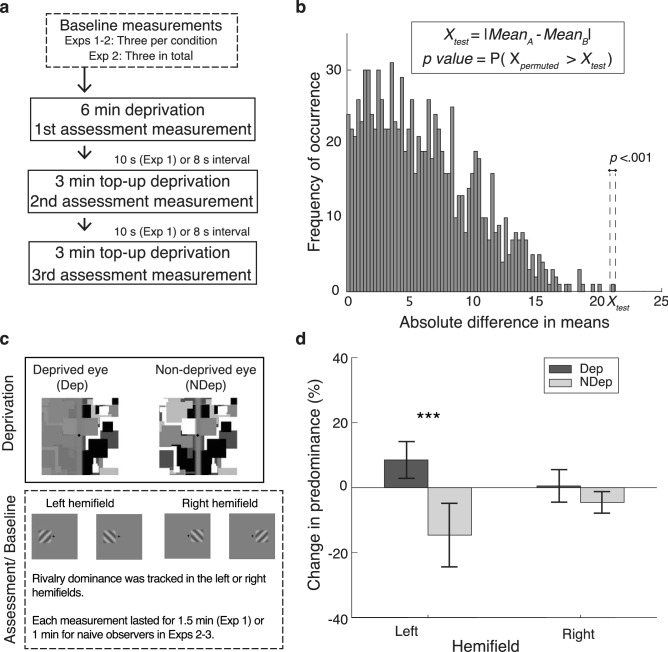
Experimental details and results of Experiment 1. (**a**) Flow chart depicting the general task procedure used across experiments. (**b**) Illustration of the permutation test. To evaluate an effect of interest, the absolute difference in condition means was computed and evaluated against an empirical distribution compiled from the permutated values of the dataset. *P* values are estimated from the proportion of permutated values larger than the test statistic, depicted as less than 0.001 in this example. (**c**) Stimulus presentation. Monocular deprivation was conducted in the left visual hemifield, after which its effect was assessed in the left or right visual hemifield. (**d**) Results of Experiment 1. Indicative of spatial specificity, significant shifts in predominance were only observed in the previously deprived left hemifield. Error bars are the 95% confidence intervals and central tendencies are represented by the group mean. Asterisks represented significance level (*p* < 0.05: *, *p* < 0.01: **, *p* < 0.001: ***).

In vision, cooperative and inhibitory interactions among neighbouring spatial zones have been observed in both binocular^29,30^ and dichoptic viewing conditions^31–36^. This raises two questions: (1) whether or not the DE is a retinotopic effect that only affects stimuli presented to deprived retinal locations, and (2) if local DEs interact with neighbouring spatial zones. To answer these questions, we first determined the spatial specificity of the DE in Experiment 1. Monocular deprivation was limited to a local area and its effect was recorded in both deprived and non-deprived locations (see sequence of events and stimuli in Fig. 1a,c). Experiment 2 then evaluated the influence of spatiotopic information, which represents the spatial relationships in the external world and draws on cues including retinal locations^37^, body-centred vestibular^38^ and proprioceptive cues^39,40^. Finally, Experiment 3 examined how local DEs affect surround suppression, a type of spatial interaction where the perceived strength of a central visual target is reduced by the presence of surrounding stimuli sharing similar orientation or motion content^32,33^. The surrounding stimuli can be presented in the same eye as the central target (monocular surround) or in the opposite eye (interocular surround), and it has been shown that the use of monocular surrounds produces greater suppression of the central target than interocular surrounds^32,33^. Similar to Kim et al.^18^, we presented a brief interocular contrast imbalance (3–6 min) and quantified the DE using rivalry predominance (but see other metrics in the Supplementary Information). This allowed us to simulate conditions in monocular occlusion^41,42^, avoid issues with feature selectivity in interocular suppression^43,44^, and obtain significant DEs with shorter periods of monocular deprivation^18^. The results revealed a retinotopic effect of brief monocular deprivation that reduced spatial inhibition from neighbouring areas interocularly and monocularly.

## Results

### Experiment 1

We first sought to determine if the effects of brief monocular deprivation are spatially specific. An interocular contrast imbalance was presented in the left hemifield, resulting in a 30% interocular difference in global RMS contrast. Ocular dominance was assessed in the left and right hemifields (Fig. 1b) and statistical evaluation was conducted using permutation tests (Fig. 1c; see also the “Methods” section). If deprivation involved spatially global processes, significant changes in predominance would be obtained in both hemifields. Conversely, significant changes will be limited to the left hemifield if local processes were involved. Results showed that the main effect of hemifield, assessed by averaging the predominance changes between the eyes and comparing the resultant values between the two hemifields, was not significant, *Bias* = − 0.32, *p* = 0.79. This was presumably due to a significant interaction between the eye of origin and hemifield, *Bias* = 0.07, *p* = 0.008. In the left hemifield, changes in predominance shifted significantly to the deprived eye (*Bias* = 0.002, *p* < 0.001), averaging at 8.5% (*SD* = 9.1%) for the deprived eye and − 14.6% (*SD* = 15.8%) for the non-deprived eye. In contrast, no significant shifts were observed in the right hemifield, with an average change of 0.5% (*SD* = 8.1%) in the deprived eye and − 4.5% (*SD* = 5.3%) in the non-deprived eye*, Bias* = 0.26, *p* = 0.12*.* These trends are indicative of a spatially specific DE and are illustrated in Fig. 1d.

### Experiment 2

**Figure 2:**
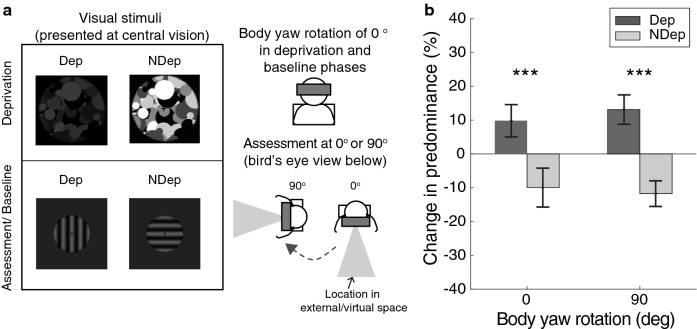
Experimental details and results of Experiment 2. (**a**) Stimulus presentation. All visual stimuli were presented in central vision, keeping the retinotopic information constant. To examine the effect of spatiotopic information, body yaw rotation was fixed or varied between deprivation and assessment phases. (**b**) Results of Experiment 2. Indicative of a retinotopic effect, significant shifts in predominance were obtained when body yaw rotation was fixed, and when body yaw rotation varied between deprivation and assessment phases. Error bars are the 95% confidence intervals and central tendencies are represented by the group mean. Asterisks represented significance level (*p* < 0.05: *, *p* < 0.01: **, *p* < 0.001: ***).

In Experiment 1, the left and right hemifields differed in retinal locations (retinotopic) *and* their respective representations of the external space (spatiotopic). Thus, the goal of Experiment 2 was to examine the influence of spatiotopic information. Body yaw rotation was manipulated between the deprivation (always at 0°, see Fig. 2a) and assessment phases (0° or 90°). During body yaw rotation, changes in spatiotopic representations were provided by vestibular and proprioceptive cues, which signalled self-movement and a change in position in external space^39^. Visual text prompts were also located at 0° and 90° in the virtual environment, and the variation in visual information that inevitably results from body yaw rotation provided further spatiotopic information. An 8-s pause was included between the end of deprivation and the onset of assessment. This lengthy pause allowed the participant ample time to reorient when a 90° rotation was required and ensured that any transient vestibular, motor or proprioceptive effects arising during active re-orienting^45,46^ would have passed before the assessment phase commenced.

Visual stimuli were always presented in central vision, and this kept the retinotopic information constant. If the effect of brief monocular deprivation were strongly influenced by spatiotopic cues, then a *fixed* body yaw rotation would produce larger shifts in predominance than a *variable* body yaw rotation. Otherwise, body yaw rotation would not affect the magnitude of predominance shifts. The results demonstrated that the DE is a retinotopic effect. As described in Fig. 2b, the eye of origin did not interact significantly with body yaw rotation, *Bias* = 0.16, *p* = 0.47 and there was no significant main effect of body yaw rotation, *Bias* = 0.31, *p* = 0.29. Pairwise comparisons showed that significant shifts in predominance were observed when body yaw rotation was fixed, averaging at 9.8% (*SD* = 8.1%) for the deprived eye and -10% (*SD* = 9.7%) for the non-deprived eye*, Bias* = 0.002, *p* < 0.001. Similar shifts were observed when body yaw rotation was varied, averaging at 13.1% (*SD* = 7.4%) for the deprived eye and − 11.7% (*SD* = 6.4%) for the non-deprived eye*, Bias* < − 0.0001, *p* < 0.001.

### Experiment 3

**Figure 3:**
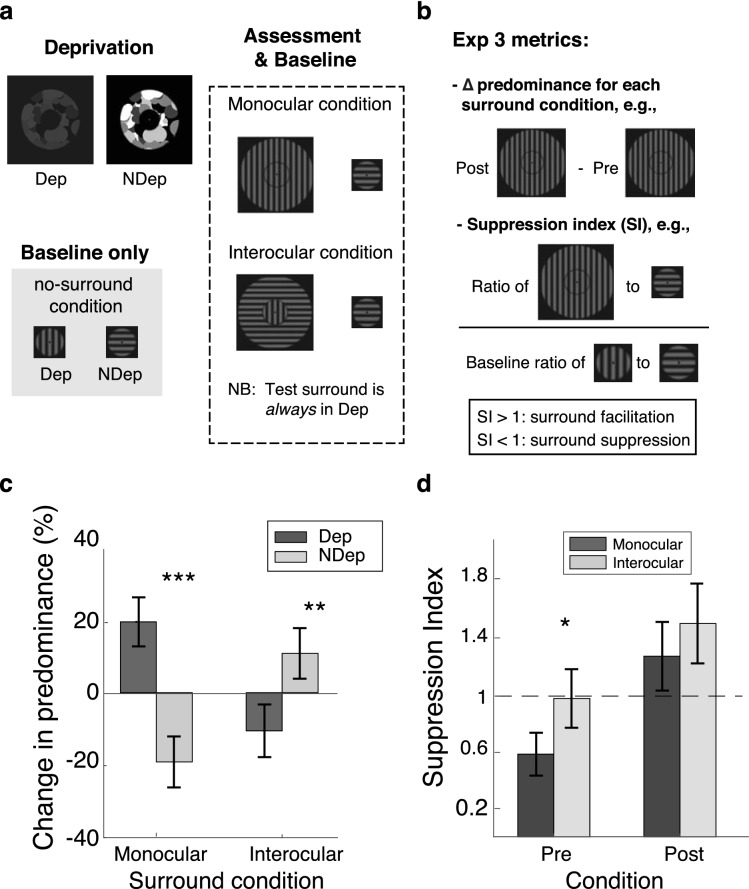
Experimental details and results of Experiment 3. (**a**) Stimulus presentation. All visual stimuli were presented in central vision, varying only in the type of experimental condition. Given the spatially specific result in Experiment 1, no-surround conditions were only assessed at baseline. Monocular and interocular surround conditions were assessed before and after deprivation. (**b**) Metrics used to evaluate the effect of monocular deprivation on surround suppression. (**c**) Effect of deprivation on the change in predominance for each surround condition. When the test surround matched the central target of the deprived eye (monocular condition), the predominance of the deprived eye increased and the predominance of the non-deprived eye decreased (standard DE). In contrast, the opposite trend was observed when the test surround matched the central target of the non-deprived eye. (**d**) Effect of deprivation on the suppression index. Pre-deprivation, suppression indices were less than 1, indicating surround suppression. The magnitude of suppression, however, was larger in the monocular condition. Post-deprivation, indices for both surround conditions were larger than 1, indicating surround facilitation. Error bars are the 95% confidence intervals and central tendencies are represented by the group mean. Asterisks represented significance level (*p* < 0.05: *, *p* < 0.01: **, *p* < 0.001: ***).

In surround suppression, presenting monocular or interocular surrounds with similar content as a central visual target reduces the perceived strength of the target^32,33^. Having established that the DE is retinotopic, Experiment 3 asked if prior monocular deprivation of the surrounding region affects the suppression of stimuli in the central, non-deprived region. To investigate, monocular deprivation was conducted within an annular region (Fig. 3a), and we compared the pre- and post-deprivation effects of test surrounds on central rivalling targets. Test surrounds were always presented to the *deprived eye* and their orientations were fixed within each rivalry measurement, matching either the orientation content of the central target in the deprived eye (monocular surround condition) or the content of the non-deprived eye (interocular surround condition). The effect of deprivation was then quantified using two metrics: (1) the change in predominance for each eye in both surround conditions, and (2) the suppression index, which estimates the impact of the test surround on the same central target (presented in the same eye) in the parallel surround and no-surround conditions (Fig. 3b; see also Methods for more details). Assuming that the DE does not alter lateral spatial interactions, we expected the strength of surround suppression to increase in both monocular and interocular surround conditions, regardless of the metric used. As depicted in Fig. 3c, we found that the type of surround condition did not significantly affect the pre- and post-deprivation changes in predominance in each eye, *Bias* = − 0.95, *p* = 0.90. However, there was a significant interaction between the eye of origin and surround condition (*Bias* = 0.003, *p* < 0.001), which interestingly, demonstrated an increase in cooperative spatial interactions. Specifically, predominance of the deprived eye increased when its target was parallel to the surround (*M*_*dep*_ = 19.1%, *SD*_*dep*_ = 8.9%) and decreased in the non-deprived eye (*M*_*Ndep*_ = − 18.7%, *SD*_*Ndep*_ = 9.3%)*, Bias* = 0.002, *p* < 0.001. The opposite trend was observed when the surround matched the target of the non-deprived eye, significantly rising its predominance (*M*_*Ndep*_ = 10.6%, *SD*_*Ndep*_ = 9.2%) and decreasing the predominance of the deprived eye (*M*_*dep*_ = − 10.3%, *SD*_*dep*_ = 9.6%)*, Bias* = 0.008, *p* = 0.001.

In the second part of the analysis, the relative dominance of an eye in a particular experimental condition (e.g., parallel surround or no-surround) was assessed by dividing its predominance with that of the contralateral eye. From these values, we derived the suppression index by dividing the values obtained for the parallel surround condition with those of the no-surround condition (see formula in “Methods” section). In short, an index of 1 represented no effect of the surround, indices smaller than 1 indicated surround suppression and indices larger than 1 represented facilitative effects. The results are described in Fig. 3d. Pre-deprivation indices were smaller than 1 in both surround conditions (*M*_*mono*_ = 0.59, *SD*_*mono*_ = 0.30 and *M*_*inter*_ = 0.98, *SD*_*inter*_ = 0.22), but the extent of surround suppression was significantly larger in the monocular condition, *Bias* = 0.08, *p* = 0.02. After deprivation, both monocular and interocular surround conditions were facilitative after deprivation, producing an average suppression index of *M*_*mono*_ = 1.28 (*SD* = 0.35) and *M*_*inter*_ = 1.52 (*SD* = 0.41), respectively*.* This main effect was found to be significant, *Bias* = 0.16, *p* = 0.04. Compared to the pre-deprivation phase, however, suppression indices did not differ significantly between the monocular condition and the interocular condition, *Bias* = 0.48, *p* = 0.30. This interaction between the type of surround condition and deprivation did not reach statistical significance, *Bias* = 0.35, *p* = 0.49.

## Discussion

The deprivation aftereffect (DE) refers to the paradoxical boost in eye dominance after short-term monocular deprivation^10,13,18^. Here, we examined the spatial specificity of the DE and its effects on the interactions between deprived areas and neighbouring, non-deprived areas. Using an interocular contrast imbalance, we selectively weakened stimulation to the left visual hemifield of the deprived eye. The DE was then measured using BR dominance tracking in both the left and right hemifields (Fig. 1c). The selective deprivation of the left hemifield resulted in a lower global stimulation in the deprived eye (i.e., interocular RMS contrast difference of 30%), but only the left hemifield showed a significant DE. We conclude this spatially specific effect is also retinotopic, as varying the body yaw rotation (and thus the spatiotopically defined location) between deprivation and assessment phases in Experiment 2 did not affect the magnitude of the DE (Fig. 2b).

Spatial representations of the external world draw on a variety of information, including retinal locations^37^, vestibular^38^ and proprioceptive cues^39,40^. It is also shown to be dependent on spatial attention, taking on a more retinotopic tuning in areas such as MT when participants simultaneously engaged in an attentionally demanding task^47^. In Experiment 2, retinotopic information was maintained by having participants attend to and track events (i.e., the fixation symbol changes and the rivalry alternations, respectively) in central vision during the deprivation and assessment phases. Spatiotopic information was manipulated through active body yaw rotation, and textual prompts were used to provide additional body-centred spatial information regarding the changes in space (see methods of Experiment 2). It is possible that a richer virtual environment with more prominent landmarks and/or less attentionally demanding task requirements, e.g., measuring relative ocular dominance using eye tracking^48,49^ would have provided more spatial cues and allowed for more spatiotopic influences on the DE. Nevertheless, evidence from previous studies suggests an early locus for the DE, linking the counterintuitive phenomenon with early visual areas such as V1^24,25,50^. As V1 is also found to be largely retinotopic regardless of spatial attentional demands^51^, modifying these conditions might have a limited effect on increasing spatiotopic influence.

The present study also examined how local DEs affect neighbouring, non-deprived areas. Specifically, in Experiment 3, we presented an interocular contrast imbalance within an annular region and studied its effects on interocular and monocular surround suppression (Fig. 3a). To generate interocular and monocular surround conditions, the test surround was always presented to the deprived eye, matching in orientation content with either the central target of the deprived eye or that of the non-deprived eye. As described in Fig. 3b, the DE was quantified in two ways. First, by computing the change in predominance for each type of surround (i.e., monocular or interocular). Second, by computing suppression indices, which compares the pre- and post-deprivation predominance values for each surround condition with that of the baseline, no-surround condition. Assuming that existing lateral spatial interactions were not affected by deprivation, local DEs were predicted to enhance surround suppression in both monocular and interocular surround conditions. Results showed that, consistent with the previous studies^32,33^, pre-deprivation central targets became less dominant when they were embedded in a parallel surround, and the decrease was greater in monocular surround condition than the interocular surround condition, i.e., 41% versus 2% decrease in suppression index (Fig. 3d). However, contrary to our predictions, post-deprivation spatial surrounds became facilitative, increasing the dominance of parallel central targets regardless of the metric used and the eye they were presented to (Fig. 3d).

We discuss the possible mechanisms underlying this pattern of results. Despite the spatially specific effect observed in Experiment 1, the spatial tuning of the DE remains unknown. With a smaller spatial separation between the deprived and non-deprived areas (i.e., 1.68° vs. 0.14°) in Experiment 3, it is plausible that the effects of deprivation might have spread to the neighbouring central area. Under this account, the neighbouring central target area in the *deprived eye* would gain a boost in predominance from the rise in sensitivity in the deprived surround area, resulting in a reduction in surround suppression. However, as any spillover effects would not produce changes in surround suppression mechanisms, any potential boost to the central target area would not be sufficient to produce the facilitation observed in the monocular surround condition. The account is also not consistent with the surround facilitation in the interocular condition, because the central target in that condition was in the *non-deprived eye* and would have decreased in dominance from any spill over effects.

Given the interocular contrast imbalance during the deprivation phase, an alternative mechanism would be contrast adaptation. With contrast adaptation, post-deprivation sensitivity towards the surround area would have increased in the deprived eye and decreased in the non-deprived eye. However, as the test surround was always presented to the *deprived eye,* its increased contrast sensitivity would have led to greater surround suppression in both interocular and monocular surround conditions. This reduction is also predicted to be larger for the interocular surround condition, as monocular surrounds have been found to be less susceptible to contrast adaptation^52,53^. As neither of these patterns were observed in our results, a pure contrast adaptation account is not an adequate explanation. Instead, it seems more likely that our findings represent a change in *lateral spatial interactions*.

Lunghi et al.^50^ found that the resting GABA concentration levels in V1 decreased after short-term monocular deprivation, and that the magnitude of reduction correlates strongly with the magnitude of the DE. This relationship between GABA levels and DE magnitudes may explain the orientation-specific, surround facilitation observed in Experiment 3, as lower GABA-mediated inhibition has been associated with weaker orientation-specific surround suppression^54–56^ and greater cortical excitability^57^. Moreover, with reduced spatial inhibition, the increased sensitivity to the deprived surround area may further promote the dominance of the central target through cooperative lateral interactions, which propagate more rapidly within collinear contours^31^. This can be contrasted with Experiment 1, where the wider spatial separation between the deprived and non-deprived spatial areas (i.e., 1.68° vs. 0.14°) and the absence of a test surround might have contributed to the lack of facilitative effects. Systematic manipulations of visual contrast and spatial separation between deprived and non-deprived areas would be required to further evaluate this account, as these properties have been found to influence the directionality and strength of lateral interactions^58,59^.

A brief deprivation period was used in this study (i.e., 3 or 6 min), raising the question of whether the underlying mechanism is identical to that underlying longer deprivation methods. Psychophysically, DEs resulting from brief deprivation periods (i.e., 15 min or less) are better captured by BR predominance than dominance duration (see other metrics in the Supplementary Information)^18^, whereas longer deprivation periods are capable of producing longer and larger distributional shifts in rivalry dominance^10^. In our view, these differences may indicate a single mechanism that gains strength over time or point to the involvement of distinct short- and long-term processes, as previously demonstrated in orientation deprivation^60^. In either scenario, a brief deprivation duration would still tap on shared mechanisms, though it is noteworthy that the degree of similarity is dependent on other determinants such as the type of visual property examined, assessment task, and stimulus content. This is because experience-dependent plasticity in binocular vision involves a multitude of possible processes^61^ and variations in these determinants may tap different neural correlates that perform differently at different time scales^62,63^.

If we take the aforementioned determinants into consideration, it becomes clear that the current brief deprivation method is unlikely to elicit vastly distinct neural correlates from longer deprivation methods. First, a rivalry dominance tracking task was used to evaluate the DE, similar to previous studies^10,18,50^. Interocular suppression processes, such as those involved in dominance tracking, have been found to implicate GABA-mediated circuits in V1^64^. These GABA-mediated mechanisms are also linked to longer deprivation effects^50^, suggesting that the current observed changes in rivalry dominance may have shared neural substrates with longer deprivation methods. Second, the current study tested for retinotopicity in the DE. As retinotopicity is a basic visual property observed in individuals with varying degrees of visual deprivation, e.g., early blind and normal sighted individuals^65^, it is unlikely to differ greatly between longer and shorter deprivation periods. Finally, previous longer deprivation methods have used pattern deprivation^10,13,15,16^. This is similar to the current method, as attenuating the contrast of the Mondrian sequence in the deprived eye would have greatly attenuated its higher spatial frequency content, given its 1/f spatial profile^66^.

Evidence from animal research suggest that GABAergic shifts are necessary for triggering ocular dominance plasticity, but these shifts are not required to maintain the changes in ocular dominance^67^. Changes in GABAergic levels also likely to be restricted between two thresholds^68^, the first of which allows ocular dominance plasticity to be expressed^69^ and the second allows the closure of the sensitive period^70^. We have suggested that a GABA-mediated account could have contributed to the surround facilitative effects in Experiment 3. Given this, increasing the duration of deprivation would enhance surround facilitative effects in the immediate post-deprivation period, but in a gradual manner akin to the small DE increase observed in Min et al. (i.e., 25% increase in magnitude over a 20-fold increase in deprivation duration)^19^. It is unclear at this point if the maintenance of these surround facilitative effects depends on GABAergic concentration or are supported by longer-term mechanisms that are activated with longer deprivation durations. Moving forward, future endeavours could consider systematic comparisons between different lengths of deprivation periods and recording the time course of GABAergic concentration changes.

Translational research exploiting the DE is underway. To list a few examples, studies have explored enhancing the DE with concurrent physical exercise, with mixed results^71,72^, but the combined use of exercise and monocular patching has been shown to improve visual acuity and recover stereopsis in adult, anisometropic amblyopia for up to a year^73^. The findings of the current study expand the application potential of the DE. For example, eye patching is a suggested treatment for spatial neglect, a condition where patients fail to detect or respond to stimuli presented contralaterally to a brain lesion^74^. The general approach is to increase ipsilesional activity (and thereby contralateral sensitivity), either through reduced inhibition with monocular patching^75^ or by improving interhemispheric balance with binocular hemifield patching^76^. Outcomes of hemifield patching were found to be more promising than monocular patching^77^, though it is conceivable that the limited visibility and poor aesthetics of either method might affect adherence to the patching procedure. Local DEs could provide a viable alternative, as each treatment session is brief and could be used to boost signals in the neglected field. Additionally, the facilitative effect of local DEs on non-deprived areas suggests that the occlusion of central vision during patching treatment is not necessary. This opens up possibilities of more user-friendly and aesthetic occlusion options such as contact lenses and special glasses, where occlusion can be placed less obtrusively around the periphery yet still generate the DE in the central vision.

## Methods

### Participants

Data was collected from 27 naïve participants from the University of Sydney (age range 18–33 years), three experienced observers (range 25–30 years) who were naïve as to the purpose of the study and author SH. Of the sample, 14 (7 females), 9 (6 females) and 3 (2 females) naïve participants took part in Experiments 1–3 respectively. Another 5 participants (4 females), including 3 experienced observers and author SH, participated in Experiments 2–3. These sample sizes accord with previous DE studies that adopted BR assessments, i.e., 6–7 participants^10,18^. All participants had normal or corrected-to-normal eyesight and normal stereovision, which was assessed with the Titmus Stereo Fly Acuity Test. Experiments were approved by the Human Research Ethics Committee of the University of Sydney (2016/662 and 2019/194) and accorded with the Declaration of Helsinki. All recruited participants provided informed consent and were either given course credit or reimbursed for their time.

### Stimuli in experiment 1

Greyscale 10 Hz dynamic sequences were presented to both eyes in all experiments. Images measured 4° by 4° in the first experiment and were composed of 256 squares (varying in length from 0.26° to 0.65°). A soft-edged vertical boundary was used to divide each image into two halves (see Fig. 1c). In the deprived eye, the left half of the image sequence was attenuated to 3% root mean square (RMS) contrast, while the other half (and the entire non-deprived eye) were set to 40% RMS contrast. The rivalry stimuli were orthogonal, 2 cycles per degree (cpd) gratings set at 40% of the maximum luminance value and windowed with 1.12° by 1.12° soft-edged square masks. Each rivalling square was located 0.84° from the fixation point, resulting in a spatial separation of 1.68° between the deprived and non-deprived areas. Background luminance was set to 50% of the maximum luminance. Stimulus presentation was conducted using PsychToolbox Version 3^78^ and a mirror stereoscope. To aid stable binocular fusion, all stimuli were enclosed with checkerboard frames measuring 4.5° by 4.5° outer dimensions and 4° by 4° inner dimensions. Stable body position was maintained using a chinrest.

### Stimuli in experiment 2

The deprivation stimuli were generated using similar image sequences as Experiment 1, with the exception that the images were circular in shape (6.3° in diameter), composed of 256 circles (0.16°–0.41° in diameter) and were not divided into halves (Fig. 1b). Rivalry stimuli were 2.46° in diameter, 2 cpd gratings set to the same contrast as Experiment 1. Unlike Experiment 1, all visual stimuli were presented in central vision. Stimulus presentation was conducted with the Unity development software and viewed with a Lenovo Explorer Mixed Reality headset. To eliminate three-dimensional cues from the Unity scene, image sequences were presented as two-dimensional (2D) videos with the same 90 Hz frame rate as the headset, against a 2D, black background. The alpha level was set to 2% for the deprived eye and 100% for the non-deprived eye. Body yaw rotation was varied with a rotatable chair.

### Stimuli in experiment 3

Deprivation in Experiment 3 (Fig. 3a) was conducted using annular image sequences (composed of 256 circles, 40% RMS contrast) that had an inner diameter of 2.6° and an outer diameter of 6.89°. The same rivalry stimuli from Experiment 2 were used to provide a screening measure and a baseline from which the effect of the surround could be estimated using suppression indices (see formula in “Analysis” section). Two surround conditions were also included, where the rivalling grating in the deprived eye was in the centre of an orthogonal or parallel 2 cpd surrounding annulus with the same dimensions as the annular image sequences, separated by a smooth gap of 0.15° between the central and surround stimuli (Fig. 3a). As in Experiment 2, the stimuli were presented in central vision, viewed with a Lenovo Explorer Mixed Reality headset. The stimuli were also enclosed by 0.5° thick checkerboard rings (~ 7.4° in diameter) to aid stable fusion. Stable body position was maintained with a non-rotatable chair.

### Procedures in experiment 1

Participants first performed a baseline rivalry dominance tracking task, during which they were instructed to press and hold a key for as long as the perceived orientation remained dominant. Three measurements were recorded for each condition, each lasting 1.5 min. After acquiring the baseline measurements, two experimental blocks of deprivation and assessment phases were presented. Each block consists of three consecutive deprivation and assessment phases. At the start of each block, participants were presented with a 6-min deprivation phase, during which they were instructed to track the colour of the fixation symbol. A 10-s wait interval followed the deprivation phase, during which participants fixated on a central countdown text. The same 1.5-min rivalry dominance tracking task was later presented, followed by another two top-up phases of 3-min deprivation and rivalry dominance tracking. The spatial location of the rivalry tracking task (left or right hemifield) was presented in counterbalanced blocks. Prior to each stage of testing (e.g., baseline), each individual completed a practice session.

### Procedures in experiment 2

The procedures in Experiment 2 were largely similar to those in Experiment 1, with some variations. To reduce participant fatigue and discomfort, rivalry dominance tasks were reduced to 1-min each and a total of three baseline measures were conducted. During each experimental block, participants tracked the shape changes of the fixation point at a body yaw rotation of 0°. Following that, participants were prompted to remain in the same or different body yaw rotation during an 8-s wait interval. Note that transient vestibular, motor and proprioceptive effects occur during active re-orienting^45,46^ but the 8-s pause was more than enough to allow these effects to pass before the assessment phase began. Two sets of text prompts were presented to provide additional body-centred spatial cues; one indicated the direction of rotation and the other located at the desired body yaw rotation. As in Experiment 1, the type of body yaw rotation was tested in counterbalanced blocks.

### Procedures in experiment 3

Similar to Experiment 2, each measurement of rivalry dominance was conducted over a period of 1-min for naïve participants. A longer duration of 1.5 min was used for experienced observers. As in Experiment 2, participants also tracked the shape changes of the fixation point during the deprivation phase. Three baseline measurements were collected for each surround condition (i.e., no-surround, monocular surround and interocular surround), and only surround conditions were tested during the post-deprivation assessment phases. Surround conditions were randomised within each experimental block, and a fixed surround orientation was always used within each 1 or 1.5 min of rivalry measurement. All baseline, deprivation and post-deprivation assessment tasks were conducted at a body yaw rotation of 0°.

### Analysis

The relative dominance of each eye was first quantified using predominance, defined as the percentage of total viewing time where an eye was dominant. To compute the predominance for each eye, the rivalry tracking data were first pooled and sorted by the eye of origin. The total dominance duration for each eye was then calculated, divided by the total viewing time and multiplied by 100. The same a priori rejection criteria as in Blake et al.^11^ were used, either (1) less than 50% of reported exclusive percepts, (2) more than 70% of predominance in one eye at baseline (without surround stimuli) or (3) did not complete experiments. Consequently, data from 4, 3 and 1 participants were excluded from Experiments 1–3, respectively. For each condition (e.g., left/right hemifield in Experiment 1, monocular/interocular surround in Experiment 3), we evaluated the DE by computing the change in predominance, obtained by subtracting the pre-deprivation predominance of each eye from the respective post-deprivation values.

As an additional metric to evaluate the strength of surround suppression in Experiment 3, the suppression index was computed using Eq. (1) below:

1 $$Suppresion\; index = \frac{Sp}{{So}} \div \frac{NSp}{{NSo}}$$

To interpret Eq. (1), first assume that a vertical grating was always presented to the left eye and a horizontal grating to the right eye. In this scenario, *Sp* refers to the predominance of the left eye when the central vertical grating was concurrently presented with a matching test surround (parallel surround condition), and *So* is the predominance of the right eye. Dividing *Sp* by *So* would therefore reflect the relative dominance of the left eye (viewing the surround-suppressed vertical grating) to that of the contralateral eye. This computation was conducted before and after monocular deprivation, yielding the pre- and post-deprivation relative dominance of the left eye in the parallel surround condition. We repeated a similar calculation with the no-surround condition, where we divided *NSp* by *NSo,* respectively defined as the predominance of the left eye to that of the right eye. Finally, to estimate the impact of the test surround on the relative dominance of the central vertical grating, we divided the resultant value from the surround condition by the non-surround condition. Note that the no-surround condition was only assessed at baseline, as we had previously observed spatially specific effects in Experiment 1. While this approach might affect the magnitude of the post-deprivation suppression indices, the general trend of the data should not change.

Given the small sample size used in the study, all effects of interest were assessed with the permutation test. The permutation test is a non-parametric method that evaluates a test statistic against an empirical distribution compiled from all possible permutations of the full dataset^79^. Under this method, the *p* value is estimated from the proportion of permutated test statistics whose absolute values are greater than that of the test statistic (Fig. 1b). In the current study, the empirical distribution was compiled with 10,000 permutations and the test statistic was the absolute difference in condition means. Condition means are defined as follows. For a comparison between the non-deprived and deprived eye within the same condition (e.g., hemifield, body yaw rotation), we defined condition means as the average change in predominance of *each* eye. In assessments of the main effects, condition means were obtained by first averaging predominance changes across the eye of origin and then computing the overall average for each condition. Finally, to test for an interaction effect, the *interocular difference* in predominance changes was first computed for each condition and the average of these differences was the condition mean. To ensure that the resultant *p* values were not driven by outlier data, we evaluated our results using the jackknife procedure. This involved conducting the permutation test repeatedly, removing one participant’s data for each repetition. If the resultant *p* value were driven by that participant’s data, then the jackknife-estimated *p* value would vary substantially when the participant is removed from the dataset. This information is quantified by jackknife bias, computed as follows:

2 $$Bias = \left( {n - 1} \right) \times \left( {\widehat{{\theta_{\left( \cdot \right)} }} - \hat{\theta }} \right)$$

where ${\widehat{\theta }}_{\left(.\right)}$ was the average jackknife-estimated *p* value and $\widehat{\theta }$ was the *p* value obtained with the full dataset.

### Ethics declaration

Experiments and methods were approved by the Human Research Ethics Committee of the University of Sydney (2016/662 and 2019/194) and were conducted according to the Declaration of Helsinki.

## Supplementary information

Supplementary Information.

## Data Availability

The datasets and MATLAB analysis codes supporting this article are accessible at https://dx.doi.org/10.17605/OSF.IO/5MS6N.
