# Supplementary material for: Brief localised monocular deprivation in adults alters binocular rivalry predominance retinotopically and reduces spatial inhibition
Source: Sci Rep. 2020 Oct 30;10:18739. doi: 10.1038/s41598-020-75252-w (PMC7603489; doi:10.1038/s41598-020-75252-w)
Supplement: Supplementary file 1 — Supplementary Information. [file 41598_2020_75252_MOESM1_ESM.docx]

**Supplementary Information**

**Brief localised monocular deprivation in adults alters rivalry predominance retinotopically and reduces spatial inhibition**

Shui’er Han^1,2*^, David Alais^1^, Hamish MacDougall^1^ & Frans A.J. Verstraten^1^

^1^ School of Psychology, University of Sydney, Sydney, Australia

^2^ Institute for Infocomm Research, Agency for Science, Technology and Research, Singapore

**
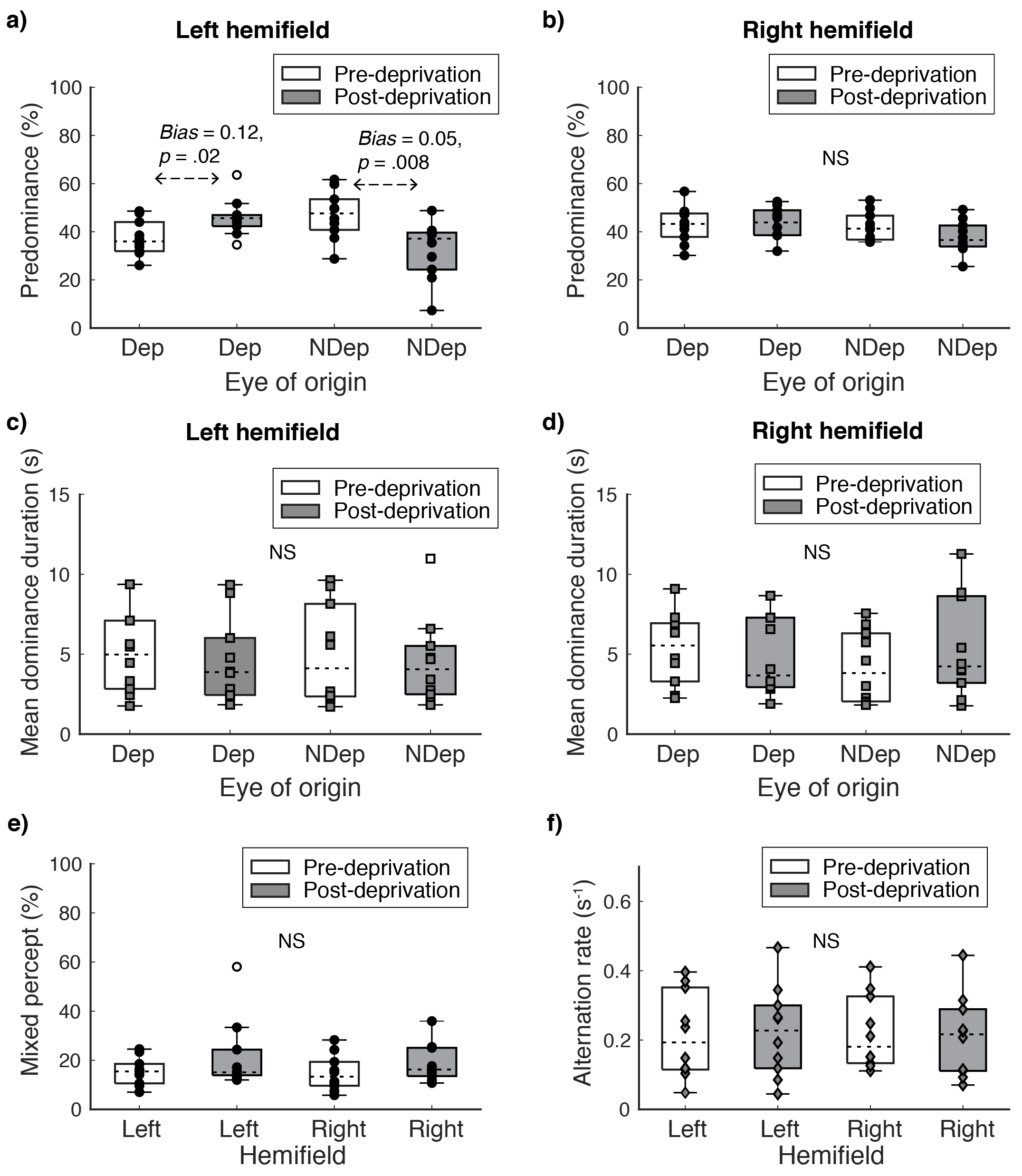
**

**Fig S1.** Supplementary results for Experiment 1. Top row: Effect of deprivation on the raw predominance values obtained for each eye, assessed by the left hemifield rivalry assessment (a) or the right hemifield rivalry assessment (b). Middle row: Pre- and post-deprivation results for mean dominance durations in the left hemifield (c) and the right hemifield (d). Bottom row: Effect of deprivation for each condition on the incidence of mixed percepts (e), estimated by subtracting the total predominance percentage across both eyes from 100, and on the rivalry alternation rate (f), estimated by dividing the total number of alternations by total viewing time within each condition. The central tendency of each metric is represented by the median, denoted as a black dashed line.

**
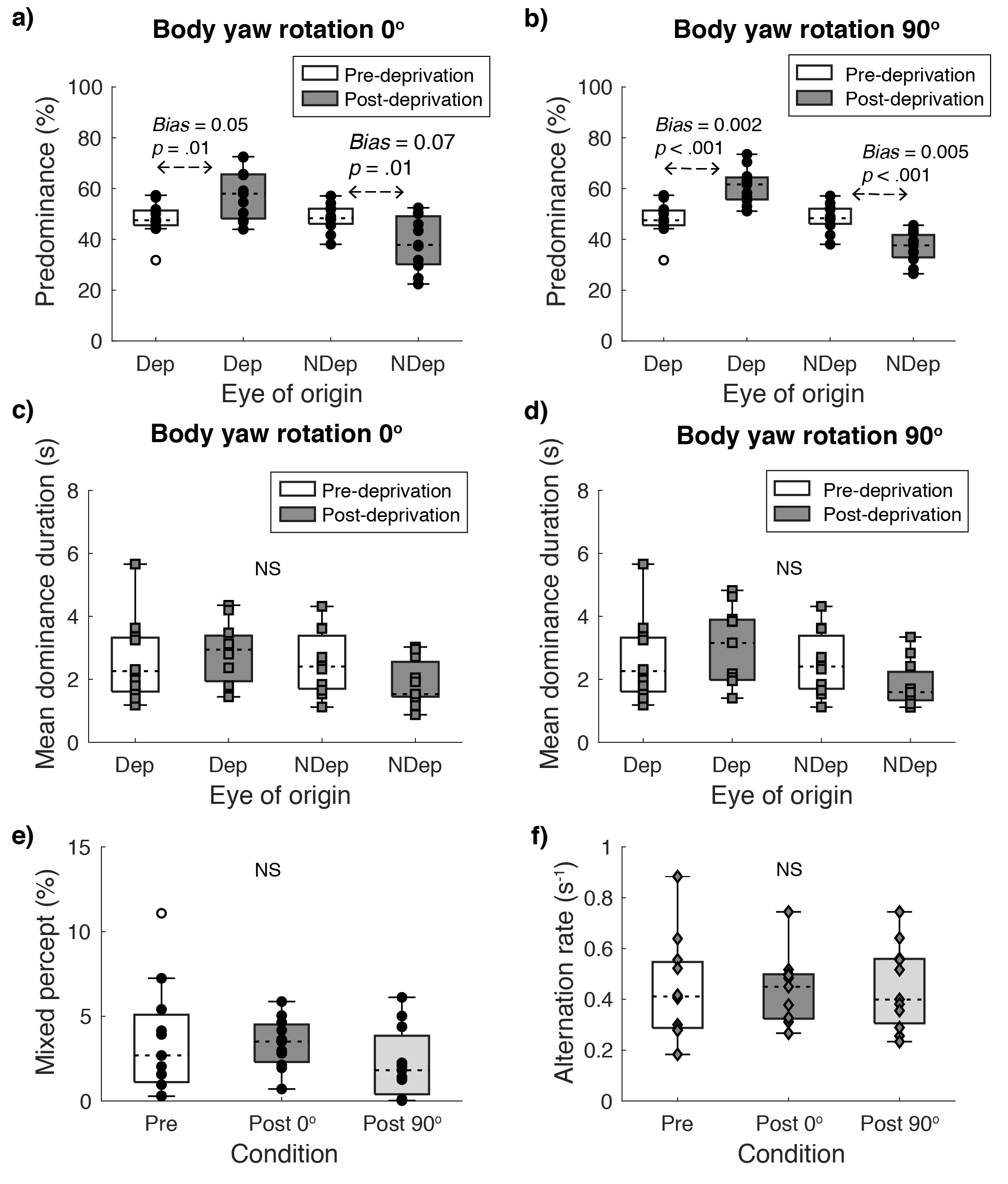
**

**Fig S2.** Supplementary results for Experiment 2. (a) Pre- and post-deprivation predominance values obtained with a fixed body yaw rotation. (b) The effect of deprivation on the raw predominance values of each eye, obtained when the body yaw rotation varied between deprivation and assessment phases. (c) Pre- and post-deprivation results for mean dominance durations collected with a fixed body yaw rotation. (d) Mean dominance durations recorded before and after deprivation with a variable body yaw rotation. e) Effect of deprivation on mixed percepts in each condition, estimated by subtracting the total predominance across both eyes from 100. (f) Rivalry alternation rate for the pre-deprivation phase and for post-deprivation condition (i.e., fixed or variable body yaw rotation). Central tendency is represented by the median, denoted as a black dashed line.

**
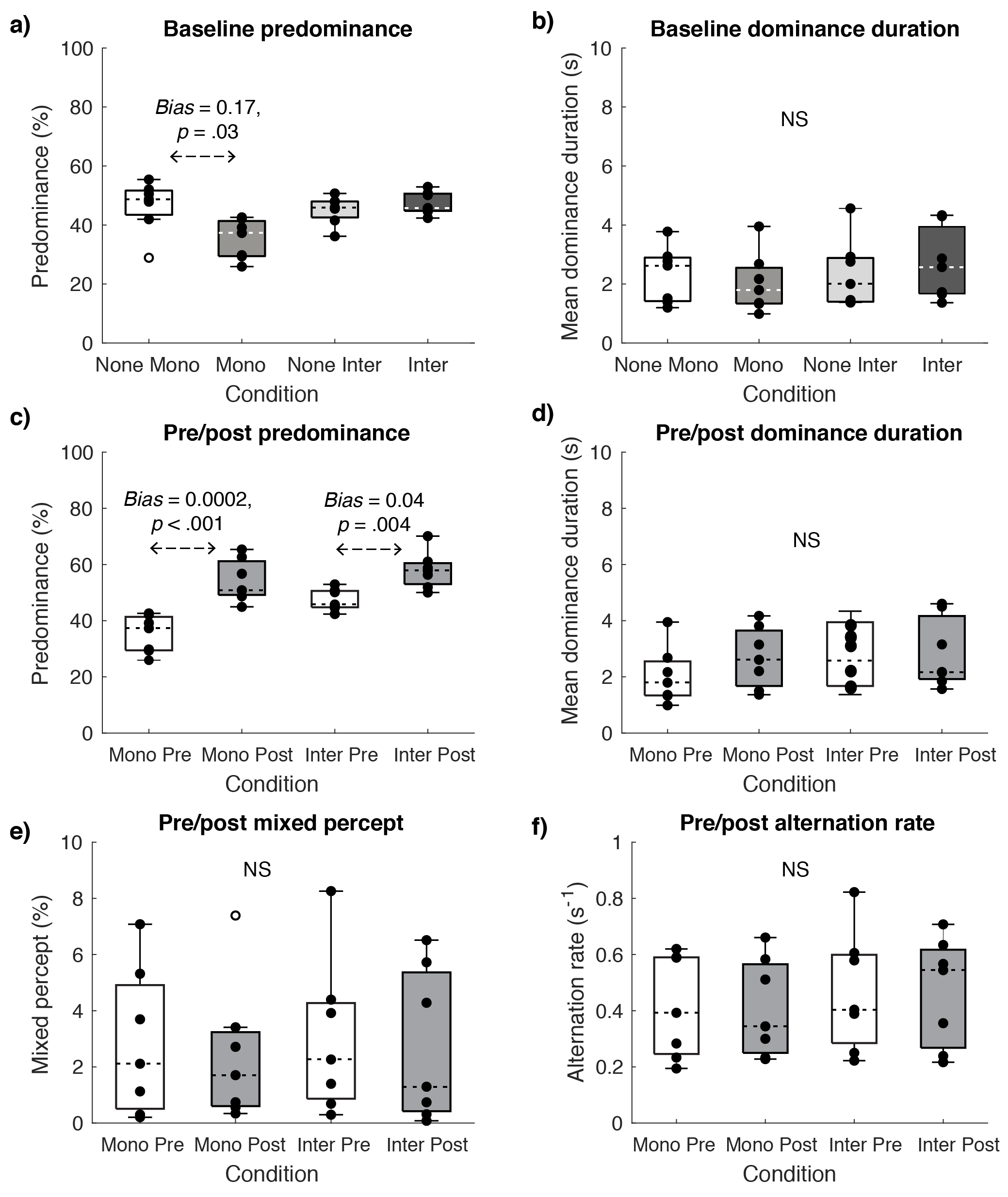
**

**Fig S3.** Supplementary results for Experiment 3. (a) Baseline predominance values for each type of central target, obtained with a parallel surround (denoted by Mono or Inter) and without surrounding stimuli (represented by None Mono and None Inter). (b) Baseline mean dominance durations for each type of central target, recorded in the presence or absence of a parallel surround. (c) Pre- and post-deprivation results for predominance values for the monocular surround condition (i.e., Mono Pre, Mono Post) and the interocular surround condition (i.e., Inter Pre, Inter Post). (d) Pre- and post-deprivation results for mean dominance durations for the monocular and interocular surround conditions. (e) The pre- and post-deprivation incidence of mixed percepts in each type of surround condition. (d) The frequency of rivalry for all pre- and post-deprivation surround conditions. As before, central tendency is represented by the median, denoted as a black or white dashed line.
